# Use of COVID-19 Vaccines for Persons Aged ≥6 Months: Recommendations of the Advisory Committee on Immunization Practices — United States, 2024–2025

**DOI:** 10.15585/mmwr.mm7337e2

**Published:** 2024-09-19

**Authors:** Lakshmi Panagiotakopoulos, Danielle L. Moulia, Monica Godfrey, Ruth Link-Gelles, Lauren Roper, Fiona P. Havers, Christopher A. Taylor, Shannon Stokley, H. Keipp Talbot, Robert Schechter, Oliver Brooks, Matthew F. Daley, Katherine E. Fleming-Dutra, Megan Wallace

**Affiliations:** ^1^National Center for Immunization and Respiratory Diseases, CDC; ^2^Vanderbilt University School of Medicine, Nashville, Tennessee; ^3^California Department of Public Health, Richmond, California; ^4^Watts Healthcare Corporation, Los Angeles, California; ^5^Institute for Health Research, Kaiser Permanente Colorado, Denver, Colorado.

**Summary**

**What is already known about this topic?**

The 2023–2024 COVID-19 vaccines provided protection against SARS-CoV-2 XBB-sublineage strains; however, these strains are no longer predominant in the United States.

**What is added by this report?**

On June 27, 2024, the Advisory Committee on Immunization Practices recommended 2024–2025 COVID-19 vaccination with a Food and Drug Administration (FDA)–authorized or approved vaccine for all persons aged ≥6 months. In August 2024, the FDA approved and authorized the Omicron JN.1 lineage (JN.1 and KP.2), 2024–2025 COVID-19 vaccines by Moderna and Pfizer-BioNTech (KP.2 strain) and Novavax (JN.1 strain).

**What are the implications for public health practice?**

The 2024–2025 COVID-19 vaccines are recommended for all persons aged ≥6 months to target currently circulating SARS-CoV-2 strains and provide additional protection against severe COVID-19–associated illness and death.

## Abstract

COVID-19 vaccination provides additional protection against severe COVID-19–associated illness and death. Since September 2023, 2023–2024 Formula monovalent XBB.1-strain COVID-19 vaccines have been recommended for use in the United States for all persons aged ≥6 months. However, SARS-CoV-2 continues to evolve, and since winter 2023–2024, Omicron JN.1 lineage strains of SARS-CoV-2, including the JN.1 strain and the KP.2 strain, have been widely circulating in the United States. Further, COVID-19 vaccine effectiveness is known to wane. On June 27, 2024, the Advisory Committee on Immunization Practices (ACIP) recommended 2024–2025 COVID-19 vaccination with a Food and Drug Administration (FDA)–approved or authorized vaccine for all persons aged ≥6 months. On August 22, 2024, FDA approved the 2024–2025 COVID-19 vaccines by Moderna and Pfizer-BioNTech (based on the KP.2 strain) for use in persons aged ≥12 years and authorized these vaccines for use in children aged 6 months–11 years under Emergency Use Authorization (EUA). On August 30, 2024, FDA authorized 2024–2025 COVID-19 vaccine by Novavax (based on the JN.1 strain) for use in persons aged ≥12 years under EUA. ACIP will continue to evaluate new evidence as it becomes available and will update recommendations as needed.

## Introduction

COVID-19 continues to account for thousands of hospitalizations and hundreds of deaths in the United States each week* (*1*). During October 2023–May 2024, U.S. COVID-19–associated hospitalization rates were highest among adults aged ≥75 years, followed by infants aged <6 months and adults aged 65–74 years (*2*). During July 2023–March 2024, among children and adolescents aged ≤17 years admitted to a hospital with COVID-19, 50% had no underlying medical conditions, with underlying conditions less common among infants aged <6 months (25%) and more common among adolescents (78%). Among hospitalized children and adolescents aged ≤17 years with COVID-19 and no underlying medical conditions, 18% were admitted to an intensive care unit. Age-adjusted COVID-19–associated hospitalization rates during October 2023–May 2024 were highest among non-Hispanic American Indian or Alaska Native persons, and non-Hispanic Black or African American persons (*1*). During May 2023–April 2024, monthly rates of COVID-19–associated death were highest among adults aged ≥75 years, followed by adults aged 65–74 years.^†^ In 2023, a total of 44,059 COVID-19–associated deaths were reported in persons aged ≥65 years, 5,634 among persons aged 20–64 years, 125 among persons aged 1–19 years, and 58 among infants aged <1 year.^§^

The 2023–2024 Formula COVID-19 monovalent vaccines were based on the XBB.1 strain; however, since winter 2023–2024, Omicron JN.1 lineage SARS-CoV-2 strains, including the JN.1 and KP.2 strains, have been widely circulating in the United States. On June 27, 2024, the Advisory Committee on Immunization Practices (ACIP) recommended 2024–2025 COVID-19 vaccination with a Food and Drug Administration (FDA)–approved or authorized vaccine for all persons aged ≥6 months. On August 22, 2024, FDA approved the 2024–2025 COVID-19 vaccines by Moderna and Pfizer-BioNTech (KP.2 strain) for use in persons aged ≥12 years and authorized these vaccines for use in children aged 6 months–11 years under Emergency Use Authorization (EUA) (*3*). On August 30, 2024, FDA authorized 2024–2025 COVID-19 vaccines by Novavax (JN.1 strain) for use in persons aged ≥12 years under EUA (*3*). ACIP’s recommendation was based on ongoing vaccine-preventable morbidity and mortality from COVID-19 in all age groups, vaccine effectiveness (VE) and safety data, cost-effectiveness, and equitable access to COVID-19 vaccine, including in disproportionately affected populations (*1*). ACIP will continue to evaluate new evidence as it becomes available and will update recommendations as necessary.

## Methods

Since June 2020, ACIP has convened 40 public meetings to review data and consider recommendations for COVID-19 vaccines.^¶^ During March–June 2024, the ACIP COVID-19 Vaccines Work Group (Work Group) met nine times to discuss the current policy question (i.e., whether 2024–2025 COVID-19 vaccination should be recommended for all persons aged ≥6 months). The Work Group used the Grading of Recommendations, Assessment, Development and Evaluation (GRADE) approach** to assess the certainty of the evidence regarding benefits and harms associated with an updated (bivalent or 2023–2024) COVID-19 vaccine administered in the United States during September 2022–May 2024. The Work Group selected this population, intervention, and period to identify evidence most applicable to what can be anticipated from the 2024–2025 COVID-19 vaccine in the United States. The Work Group used the Evidence to Recommendations framework^††^ to guide their considerations and reviewed data on the importance of COVID-19 as a public health problem and issues of resource use, benefits and harms, patients’ values, acceptability, feasibility, and equity related to COVID-19 vaccines (https://www.cdc.gov/vaccines/acip/recs/grade/covid-19-2024-2025-6-months-and-older-etr.html).

## Vaccine Effectiveness and Safety

Published assessments of VE and safety of previous COVID-19 vaccine formulations were evaluated using GRADE to assess the confidence (high, moderate, low, or very low) that the actual effect lies close to that of the estimated effect (*1*). A body of evidence that includes only randomized controlled trials begins at high certainty, whereas a body of evidence that includes observational data begins at low certainty.

The benefits of the updated (bivalent or 2023–2024) COVID-19 vaccines compared to no updated vaccination among adolescents and adults were assessed by reviewing pooled VE data for three outcomes: 1) medically attended COVID-19,^§§^ 2) COVID-19–associated hospitalization, and 3) COVID-19–associated death. Pooled VE against medically attended COVID-19 was 43% (95% CI = 30%–54%), against COVID-19–associated hospitalization was 44% (95% CI = 34%–52%), and against COVID-19–associated death was 23% (95% CI = 8%–36%) (*1*). The certainty assessment for all three outcomes was low. For infants and children, one study examining medically attended COVID-19 was available, with a VE of 80% (95% CI = 42%–96%), with a low certainty assessment. No published studies were available to assess updated VE against COVID-19–associated hospitalization and death among infants and children; therefore, benefits were inferred from adolescent and adult data. These outcomes had a certainty assessment of very low resulting from serious concern for indirectness due to the inference from data collected among a different population. The certainty assessment for prespecified adverse events (i.e., myocarditis or pericarditis and anaphylaxis) remained low for adults and adolescents and very low for infants and children. The GRADE evidence profile is available at https://www.cdc.gov/vaccines/acip/recs/grade/covid-19-2024-2025-6-months-and-older.html.

ACIP also reviewed additional, updated CDC data on VE of a 2023–2024 COVID-19 vaccine dose compared with no 2023–2024 vaccination^¶¶^ (*4*). During October 2023–April 2024, VE among adults aged ≥18 years against symptomatic SARS-CoV-2 infection 60–119 days after vaccination was 58% (95% CI = 33%–73%) for likely XBB-sublineage infection and 37% (95% CI = 13%–51%) for likely JN.1-sublineage infection. During September 2023–May 2024, VE against COVID-19–associated hospitalization among adults aged ≥18 years without immunocompromising conditions was 49% (95% CI = 43%–55%) 7–59 days after 2023–2024 vaccination, declining to 14% (95% CI = 0%–27%) 120–179 days after vaccination. As with previous COVID-19 vaccine formulations (*5*), VE against critical illness appeared somewhat more durable, at 69% (95% CI = 57%–78%) 7–59 days after 2023–2024 vaccination and 32% (95% CI = 0%–53%) 120–179 days after vaccination. Data for children and adolescents were limited, although VE was similar against medically attended COVID-19 in children and adults.

ACIP also reviewed additional CDC data on 2023–2024 COVID-19 vaccine safety. Vaccine Safety Datalink (VSD) surveillance for prespecified outcomes of special interest identified two statistical signals for mRNA COVID-19 vaccines during the 2023–2024 season (*6*). The first was for Guillain-Barré syndrome (GBS) among persons aged ≥65 years. An association between GBS and mRNA COVID-19 vaccines had not been identified before 2023–2024, and evidence as to whether this 2023–2024 signal represents an actual risk is inconclusive. In addition, VSD identified a statistical signal for ischemic stroke among adults aged ≥50 years. A similar signal had previously been observed for the bivalent COVID-19 vaccine formulation and was reviewed by ACIP in October 2023 (*7*). The cumulative data to date have not provided clear and consistent evidence of a safety problem for ischemic stroke, and a follow-up VSD study is in progress to further assess the risk for ischemic stroke after mRNA vaccination. Any real or theoretical risk of vaccine adverse events needs to be placed in the context of benefits of COVID-19 vaccines in preventing COVID-19 and its potentially serious complications, including stroke.

## Economic Analyses

Economic modeling demonstrated that COVID-19 vaccines are most cost-effective in adults aged ≥65 years, who experience the highest rates of severe COVID-19 (*8*). The base case incremental cost-effectiveness ratio (ICER) in this age group was $23,308 per quality adjusted life year (QALY) and was robust to parameter input assumptions. ICERs were $113,248 per QALY for adults aged 50–64 years and $212,225 per QALY for adults aged 18–49 years and were sensitive to input assumptions. ICERs were $202,621 in adolescents aged 12–17 years and $200,445 in children aged 5–11 years, and were highly sensitive to input assumptions (i.e., more uncertain). ICERs in persons aged <65 years were more favorable when input assumptions were varied to consider higher vaccine impact, higher risk for COVID-19–associated hospitalization, higher quality of life impact for symptomatic illness, and lower vaccine cost.

## Recommendations for 2024–2025 COVID-19 Vaccination

On June 27, 2024, ACIP recommended 2024–2025 COVID-19 vaccination with an FDA-approved or authorized vaccine for all persons aged ≥6 months.*** This recommendation includes FDA-licensed or authorized Omicron JN.1 lineage (JN.1 and KP.2) monovalent COVID-19 vaccines (i.e., Moderna and Pfizer-BioNTech [KP.2 strain] or Novavax [JN.1 strain] 2024–2025 COVID-19 vaccines), consistent with FDA-licensed indications or EUA. Because the 2024–2025 Novavax COVID-19 vaccines for persons aged ≥12 years and all 2024–2025 COVID-19 vaccines for children aged 6 months–11 years are authorized under EUA, recommendations for 2024–2025 Novavax vaccine and all 2024–2025 COVID-19 vaccines in children aged 6 months–11 years are interim recommendations. 

### Recommendations for Persons Without Moderate or Severe Immunocompromise

Persons aged 5–11 years without moderate to severe immunocompromise need 1 dose of 2024–2025 COVID-19 vaccine (Moderna or Pfizer-BioNTech) to be up to date. Persons aged ≥12 years without moderate to severe immunocompromise need 1 dose of 2024–2025 COVID-19 vaccine (Moderna, Novavax, or Pfizer-BioNTech) to be up to date (Table 1). Persons aged ≥12 years who have not previously received any COVID-19 vaccines and choose to get Novavax should receive 2 doses of the 2024–2025 Novavax vaccine. Children aged 6 months–4 years are recommended to receive an initial multidose vaccination series when they first receive COVID-19 vaccination and thus need more than 1 COVID-19 vaccine dose, including at least 1 dose of the 2024–2025 COVID-19 vaccine, to be up to date (Table 2).

**TABLE 1:** Recommended 2024–2025 COVID-19 vaccination schedule for persons aged ≥5 years who are not moderately or severely immunocompromised,* by previous COVID-19 vaccination history — United States, September 2024

Previous COVID-19 vaccination history^†,§^	2024–2025 COVID-19 vaccine	No. of 2024–2025 doses indicated	Interval between doses
**Unvaccinated**	Moderna	1	NA
	or Pfizer-BioNTech	1	NA
	or Novavax (aged ≥12 yrs only)	2	3–8 wks between dose 1 and dose 2
**Previously received ≥1 COVID-19 vaccine dose^¶^**	Moderna	1	≥8 wks after last dose
	or Pfizer-BioNTech	1	≥8 wks after last dose
	or Novavax (aged ≥12 yrs only)	1	≥8 wks after last dose

**Abbreviation:** NA = not applicable.

* Additional clinical considerations, including detailed schedules and tables by age and vaccination history for those who are and are not moderately or severely immunocompromised, are available at https://www.cdc.gov/vaccines/covid-19/clinical-considerations/covid-19-vaccines-us.html.

^†^ Before 2024–2025 vaccine.

^§^ https://www.cdc.gov/vaccines/covid-19/clinical-considerations/interim-considerations-us.html#not-immunocompromised

^¶^ Including at least 1 dose Moderna, Pfizer-BioNTech, Janssen (Johnson & Johnson) (aged ≥18 years only) COVID-19 vaccines, or at least 2 doses of Novavax COVID-19 vaccine (aged ≥12 years). Persons who received only 1 dose of Novavax (aged ≥12 years) COVID-19 vaccine should receive dose 2 of Novavax 3–8 weeks after dose 1, or if more than 8 weeks have elapsed since receipt of dose 1 of Novavax, any 2024–2025 COVID-19 vaccine (i.e., Moderna, Novavax, or Pfizer-BioNTech) may be administered.

**TABLE 2:** Recommended COVID-19 vaccination schedule for children aged 6 months–4 years who are not moderately or severely immunocompromised,* by previous COVID-19 vaccination history — United States, September 2024

Previous COVID-19 vaccination history^†,§^	2024–2025 COVID-19 vaccine	No. of 2024–2025 doses indicated	Interval between doses
**Unvaccinated**	Moderna	2	4–8 wks between dose 1 and dose 2
	or Pfizer-BioNTech	3	3–8 wks between dose 1 and dose 2 ≥8 wks between dose 2 and dose 3
**Previously received Moderna vaccine**			
1 dose any Moderna	Moderna	1	4–8 wks after dose 1
≥2 doses any Moderna	Moderna	1	≥8 wks after last dose
**Previously received Pfizer-BioNTech vaccine**			
1 dose any Pfizer-BioNTech	Pfizer-BioNTech	2	3–8 wks between dose 1 and dose 2 ≥8 wks between dose 2 and dose 3
2 doses any Pfizer-BioNTech	Pfizer-BioNTech	1	≥8 wks after dose 2
≥3 doses any Pfizer-BioNTech	Pfizer-BioNTech	1	≥8 wks after last dose

* Additional clinical considerations, including detailed schedules and tables by age and vaccination history for those who are and are not moderately or severely immunocompromised, are available at https://www.cdc.gov/vaccines/covid-19/clinical-considerations/covid-19-vaccines-us.html.

^†^ Before 2024–2025 mRNA vaccine.

^§^ https://www.cdc.gov/vaccines/covid-19/clinical-considerations/interim-considerations-us.html#not-immunocompromised

### Recommendations for Persons Who Are Moderately or Severely Immunocompromised

Persons aged ≥6 months who are moderately or severely immunocompromised should receive at least 1 dose of 2024–2025 COVID-19 vaccine. Depending on vaccination history, additional doses may be recommended. Unvaccinated persons aged 6 months–11 years who are moderately or severely immunocompromised are recommended to receive an initial 3-dose vaccination series of a 2024–2025 mRNA COVID-19 vaccine, with all doses from the same manufacturer. Unvaccinated persons aged ≥12 years who are moderately or severely immunocompromised should complete an initial vaccination series with either 3 doses of a 2024–2025 mRNA COVID-19 vaccine from the same manufacturer or 2 doses of 2024–2025 Novavax COVID-19 vaccine.

Persons who are moderately or severely immunocompromised, have completed an initial series, and have received at least 1 dose of a 2024–2025 COVID-19 vaccine, may receive 1 additional age-appropriate dose of 2024–2025 COVID-19 vaccine at least 2 months after the last recommended 2024–2025 vaccine dose. Further additional doses may be administered, guided by the clinical judgment of a health care provider and personal preference and circumstances. Any further additional doses should be administered at least 2 months after the last 2024–2025 COVID-19 vaccine dose. Additional clinical considerations, including detailed schedules and tables by age and vaccination history for persons who are and are not moderately or severely immunocompromised, are available at https://www.cdc.gov/vaccines/covid-19/clinical-considerations/covid-19-vaccines-us.html.

### Implementation Considerations

Since 2023, COVID-19 vaccines have been distributed in the commercial marketplace. The Affordable Care Act requires insurers to cover all ACIP routinely recommended vaccines without cost-sharing by the next coverage year after recommendations are made.^†††^ Section 3203 of the Coronavirus Aid, Relief, and Economic Security Act expedites coverage of COVID-19 vaccines beyond that which is required for most preventive services. COVID-19 vaccines are also covered under Medicare part B and for nearly all Medicaid beneficiaries without cost-sharing. COVID-19 vaccines are included in the Vaccines for Children Program,^§§§^ which provides vaccines to approximately one half of U.S. persons aged <19 years at no cost. CDC’s Bridge Access Program^¶¶¶^ provided free 2023–2024 COVID-19 vaccines to adults without health insurance and adults whose insurance did not cover all COVID-19 vaccine costs. However, the Bridge Access Program ended in August 2024 and will not be available to cover the 2024–2025 COVID-19 vaccine. Before vaccination, providers should provide the EUA Fact Sheet (*3*), manufacturer’s package insert, or Vaccine Information Statement regarding the vaccine being administered and counsel vaccine recipients about expected systemic and local adverse reactions (reactogenicity).

### Reporting of Adverse Events

Adverse events after vaccination should be reported to the Vaccine Adverse Event Reporting System (VAERS). For licensed COVID-19 vaccines administered to persons aged ≥12 years, reporting is encouraged for any clinically significant adverse event, even when a causal association between the vaccine and the event is uncertain, as well as for vaccination errors. For COVID-19 vaccines given under Emergency Use Authorization,**** vaccination providers are required to report certain adverse events to VAERS. Additional information is available at https://vaers.hhs.gov or by telephone at 1-800-822-7967.

## Footnotes

* https://covid.cdc.gov/covid-data-tracker/#datatracker-home (Accessed June 17, 2024).

† https://covid.cdc.gov/covid-data-tracker/#demographicsovertime (Accessed June 17, 2024).

§ https://wonder.cdc.gov/mcd-icd10-provisional.html (Accessed June 5, 2024).

¶
https://www.cdc.gov/acip-recs/hcp/vaccine-specific/covid-19.html

**
https://www.cdc.gov/vaccines/acip/recs/grade/about-grade.html

††
https://www.cdc.gov/vaccines/acip/recs/grade/downloads/acip-evidence-recs-framework.pdf

§§ Medically attended COVID-19 was defined as an emergency department or urgent care visit.

¶¶ Persons who received no 2023–2024 COVID-19 vaccine regardless of COVID-19 vaccination history (i.e., includes both previously unvaccinated and vaccinated with earlier formulations of COVID-19 vaccines).

*** ACIP voted (11 to zero with one abstention) to recommend vaccination with 2024–2025 COVID-19 vaccines as authorized or approved for persons aged ≥6 months.

†††
https://www.law.cornell.edu/uscode/text/42/300gg-13

§§§
https://www.cdc.gov/vaccines-for-children/about/index.html

¶¶¶
https://archive.cdc.gov/#/details?url=https://www.cdc.gov/vaccines/programs/bridge/index.html

**** 2024–2025 COVID-19 vaccines that are under FDA EUA are Moderna and Pfizer-BioNTech for use among children aged 6 months–11 years, and Novavax for use among persons aged ≥12 years.
